# Conversion surgery for hepatocellular carcinoma with portal vein tumor thrombus after successful atezolizumab plus bevacizumab therapy: a case report

**DOI:** 10.1186/s12957-022-02691-2

**Published:** 2022-07-12

**Authors:** Yoshifumi Hidaka, Miyo Tomita, Ryosuke Desaki, Masahiro Hamanoue, Sonshin Takao, Mari Kirishima, Takao Ohtsuka

**Affiliations:** 1 Department of Surgery, Tanegashima Medical Center, 7463 Nishinoomote, Nishinoomote, Kagoshima 891-3198 Japan; 2 Department of Pathology, Field of Oncology, Kagoshima University Graduate School of Medical and Dental Sciences, Kagoshima University, 8-35-1 Sakuragaoka, Kagoshima, 890-8520 Japan; 3 Department of Digestive Surgery, Breast and Thyroid Surgery, Graduate School of Medical and Dental Science, Kagoshima University, 8-35-1 Sakuragaoka, Kagoshima, 890-8520 Japan

**Keywords:** Unresectable hepatocellular carcinoma, Portal vein tumor thrombus, Atezolizumab plus bevacizumab therapy, Conversion surgery, Case report

## Abstract

**Background:**

The treatment of hepatocellular carcinoma (HCC) requires diverse and multidisciplinary approaches. In recent years, new agents with good antitumor effects have emerged for systemic chemotherapy, and conversion surgery (CS) after systemic chemotherapy is expected to be an effective treatment strategy for unresectable HCC. We herein report a case of unresectable HCC with portal vein tumor thrombus (PVTT) in which atezolizumab plus bevacizumab therapy induced PVTT regression, followed by CS with R0 resection.

**Case presentation:**

The patient was a 79-year-old man with S2/S3 HCC who was referred to our department due to tumor re-growth and PVTT after two rounds of transcatheter arterial chemoembolization. The PVTT extended from the left portal vein to the main trunk, and it was determined that the resection of the left portal vein would be difficult to perform with R0 status. Based on the diagnosis of unresectable HCC, treatment with atezolizumab plus bevacizumab was initiated. After two courses of treatment, contrast-enhanced computed tomography showed that the PVTT had regressed to the peripheral side of the left portal vein, and R0 resection became possible. The patient developed grade 3 skin lesions as an immune-related adverse event, and it was determined that the continuation of chemotherapy would be difficult. Four weeks after the second course of atezolizumab plus bevacizumab administration, left lobectomy was performed. Intraoperative ultrasonography was used to confirm the location of the tumor thrombus in the left portal vein during the resection, and a sufficient surgical margin was obtained. The histopathological findings showed that primary tumor and PVTT were mostly necrotic with residues of viable tumor cells observed in some areas. The liver background was determined as A1/F4 (new Inuyama classification). The resection margins were negative, and R0 resection was confirmed. There were no postoperative complications. No recurrence was observed as of five months after surgery.

**Conclusions:**

CS with atezolizumab plus bevacizumab therapy has potential utility for the treatment of unresectable HCC with PVTT.

## Background

The treatments for hepatocellular carcinoma (HCC), including hepatectomy, radiofrequency ablation, transcatheter arterial chemoembolization (TACE), and systemic chemotherapy are diverse and multidisciplinary. The portal vein tumor thrombus (PVTT) is a strong postoperative prognostic factor for hepatectomy, and PVTT at or near the main trunk or contralateral branch is generally considered to be inoperable or difficult for hepatectomy. In contrast, if the PVTT is reduced and R0 resection becomes feasible after prior systemic chemotherapy, conversion surgery (CS) may become possible.

Sorafenib has been used for standard systemic chemotherapy; however, its antitumor effect is limited [1]. In recent years, chemotherapy agents with good treatment outcomes such as lenvatinib [2] and atezolizumab plus bevacizumab [3] have emerged. These agents have demonstrated superior antitumor efficacy to that of sorafenib. Therefore, CS for unresectable HCC preceded by systemic chemotherapy with one of these agents may be a promising treatment strategy. There are a limited number of case reports on CS with lenvatinib [4–7], and atezolizumab plus bevacizumab [8, 9]. However, to our knowledge, there are no case reports on CS with atezolizumab plus bevacizumab for HCC with PVTT.

We herein report a case of unresectable HCC with PVTT in which atezolizumab plus bevacizumab therapy induced PVTT regression, followed by CS with R0 resection.

## Case presentation

**Fig. 1:**
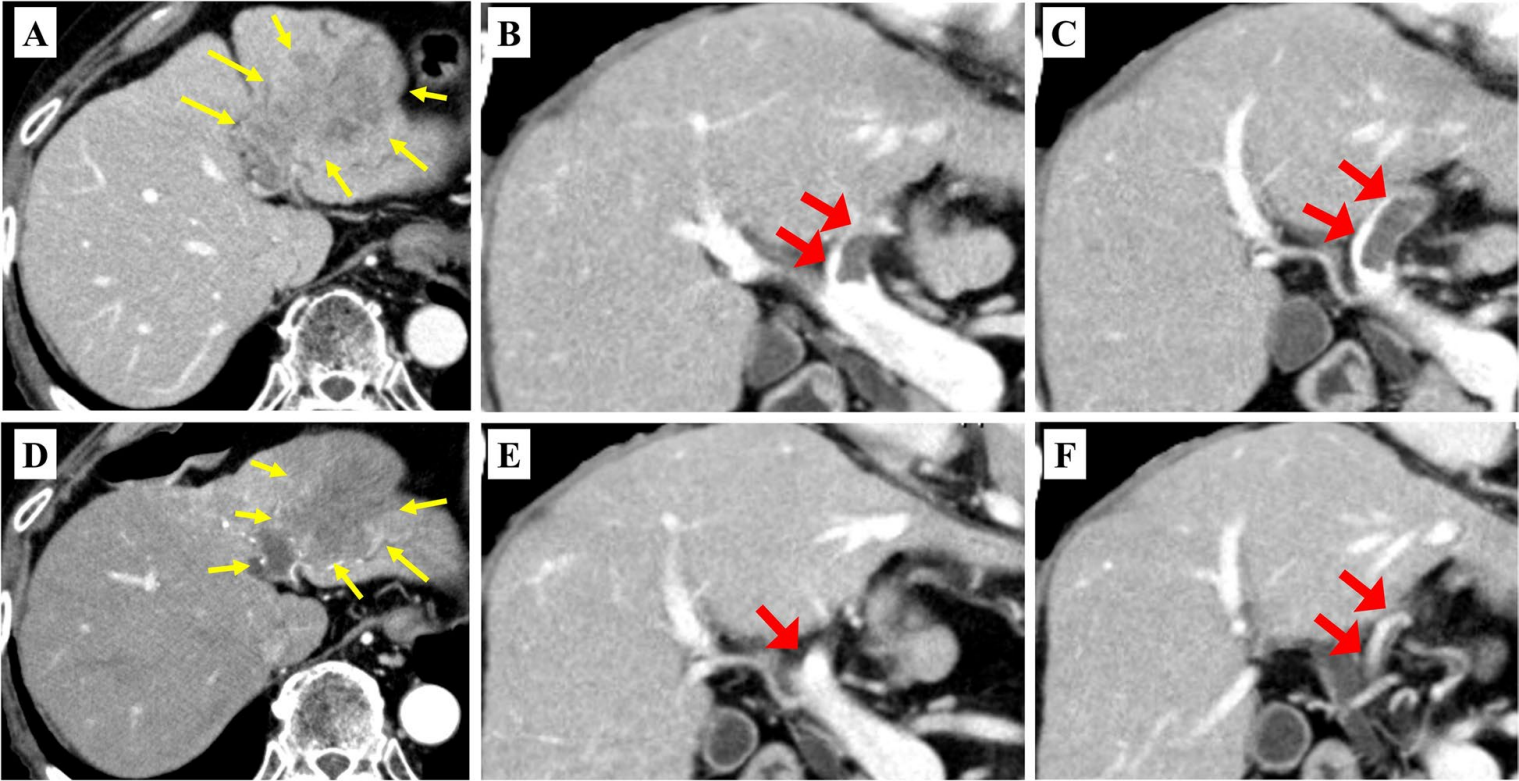
Contrast-enhanced CT findings. **A** Neoplastic lesion in S2/3 (yellow arrows). **B**, **C** Tumor thrombus from the left portal vein to the main trunk (red arrows). **D** Decreased extent of intratumoral arterial enhancement (yellow arrows). **E** The PVTT in the portal trunk regressed to the peripheral side (red arrow). **F** Reperfusion of blood flow was observed at the central portion of the left portal vein (red arrows). CT, computed tomography; PVTT, portal vein tumor thrombosis

**Fig. 2:**
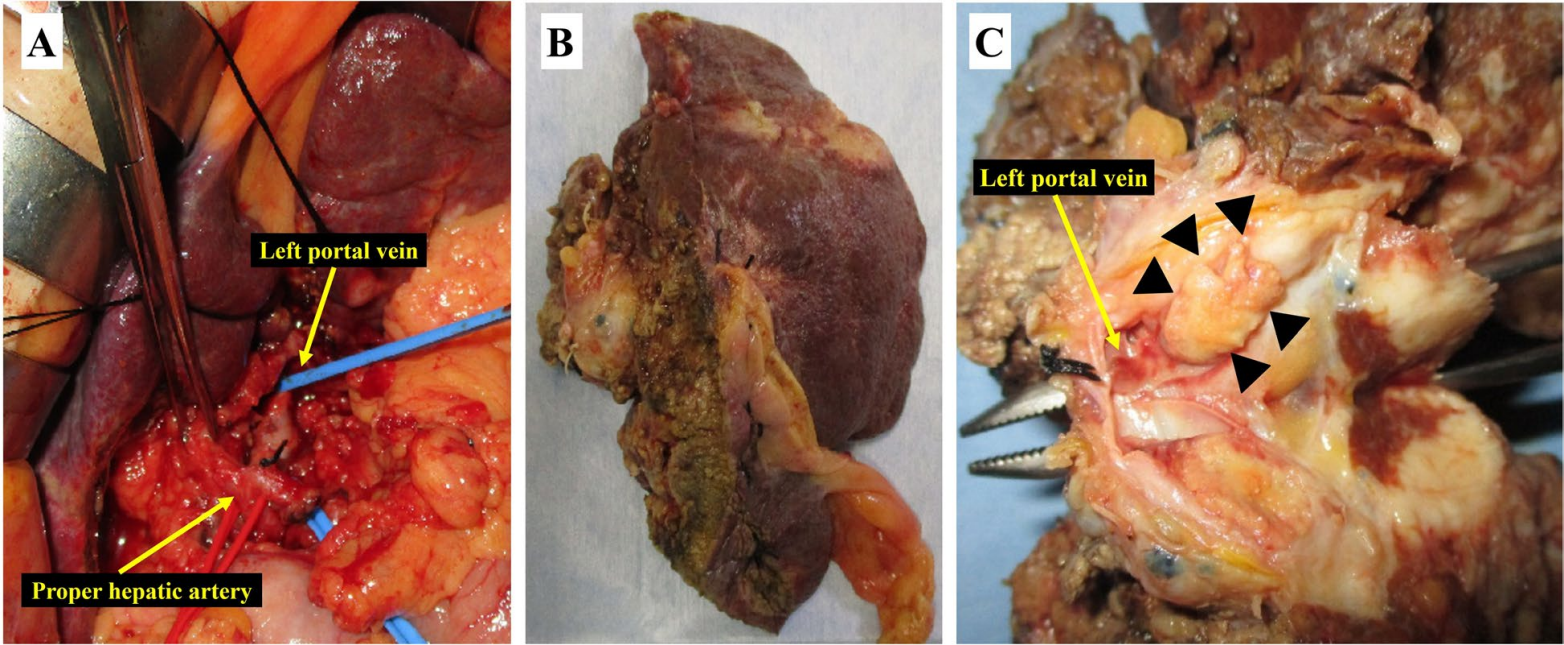
**A** Surgical findings. **B** Resected specimen. **C** Tumor thrombus (arrowhead) located approximately 1.5 cm from the resection line of the left portal vein

**Fig. 3:**
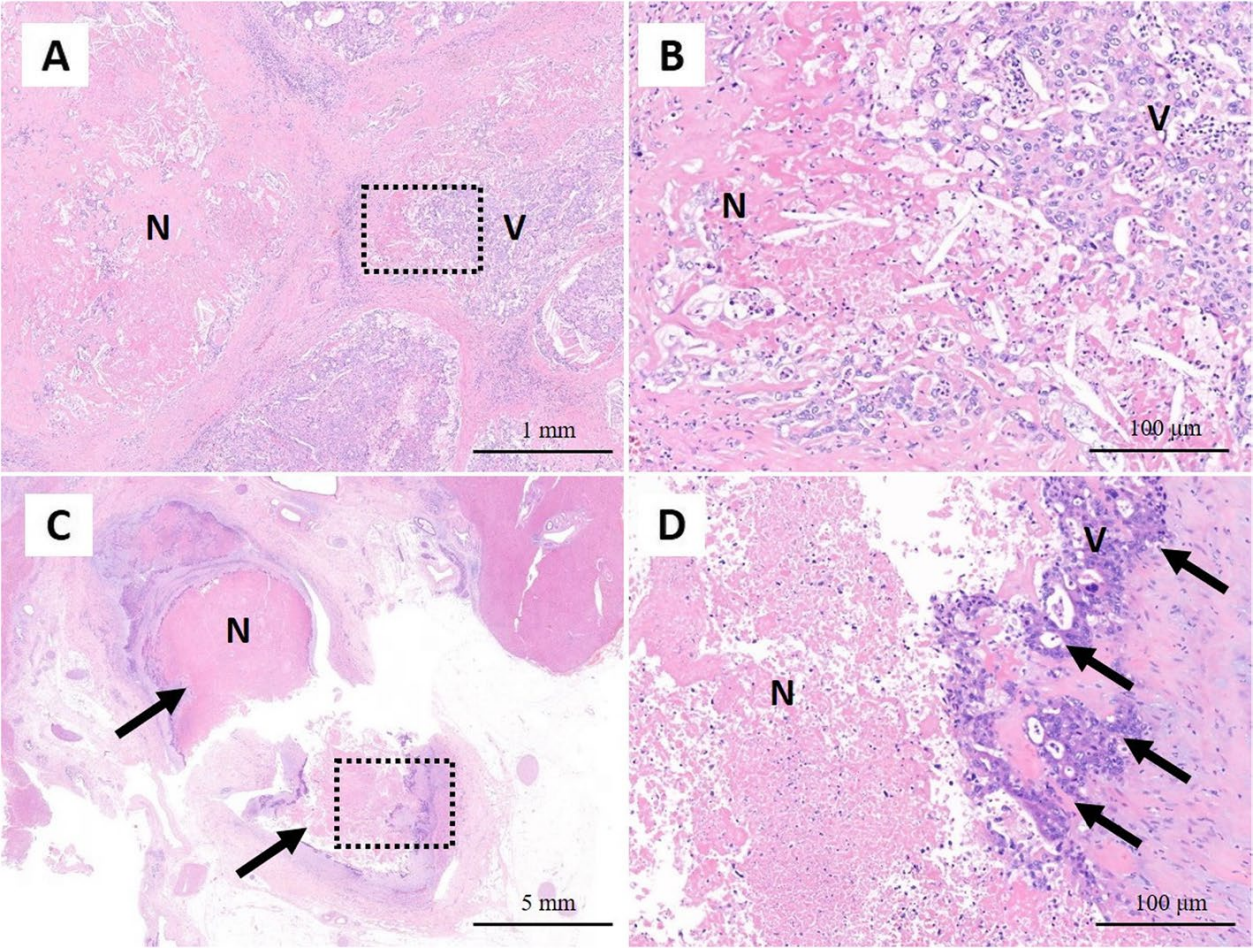
Pathological findings. Hematoxylin and eosin staining. **A** Primary tumor composed of viable cancer cells (V) with necrosis (N). **B** At a higher magnification, the infiltration of inflammatory cells and necrosis of cancer cells were observed. **C** Portal vein tumor thrombus (arrows). The majority of the thrombotic cancer cells are necrotic. **D** At a higher magnification, the residual cancer cells were observed (arrows)

A 79-year-old man was referred to our department for TACE-refractory HCC in the S2/3 area after two TACE treatments failed to control the disease. In August 2021, the contrast-enhanced computed tomography (CT) showed re-enlargement of the TACE-treated area of the S2/3 (Fig. 1A) and appearance of PVTT (Fig. 1B, C). The PVTT extended from the left portal vein to the main trunk, and it was determined that the resection of the left portal vein would be difficult to perform with R0 status. The patient was diagnosed with unresectable advanced HCC of stage IIIB (T 4 N 0 M 0) based on the eighth edition of the Union for International Cancer Control. Alpha-fetoprotein (AFP) and des-gamma-carboxyprothrombin (DCP) levels were 3.2 ng/ml and 20 mAU/ml, respectively. The patient’s condition was graded as follows: Barcelona clinic liver cancer stage C, Child-Pugh class A (6 points), and Eastern Cooperative Oncology Group Performance Status 0. He was started on a 3-week course of atezolizumab (1200 mg) plus bevacizumab (15 mg/kg) therapy. Contrast-enhanced CT after the second course showed reduced extent of intratumoral arterial enhancement without reduction of tumor size (Fig. 1D), and the PVTT had regressed from the main trunk to the peripheral side of the left portal vein (Fig. 1E, F). The patient was diagnosed with the stable disease based on the response evaluation criteria for solid tumors (RECIST), and partial response to the modified RECIST. R0 resection of the left portal vein was considered feasible with enough surgical margin. At the same time, the patient developed a grade 3 skin disorder as an immune-related adverse event, and it was judged that continuation of chemotherapy would be difficult. His tumor marker levels remained within normal range (AFP 6.4 ng/ml and DCP 24 mAU/ml). With the consent of the patient, hepatectomy was planned. Preoperative liver function was graded as Child-Pugh class A (6 points). CT volumetry showed a liver volume of 861 ml, an effective liver resection rate of 19.6%, and a residual liver volume of 692 ml. Four weeks after the second course of atezolizumab plus bevacizumab administration, in October 2021 left lobectomy was performed. During the left portal vein resection (Fig. 2A), intraoperative ultrasonography was used to confirm the location of the tumor thrombus, and a sufficient surgical margin was obtained. The duration of surgery was 5 h 43 min, blood loss was 2030 ml, and intraoperative and postoperative blood transfusions (red blood cells: 6 units and fresh frozen plasma: 10 units) were performed. The resected specimen showed that the left lobe of the liver was atrophic owing to TACE and the PVTT (Fig. 2B). The PVTT was located approximately 1.5 cm from the resection line of the left portal vein (Fig. 2C). The pathological diagnosis was moderately differentiated HCC. The histopathological findings showed that the primary tumor (Fig. 3A, B) and the PVTT (Fig. 3C, D) were mostly composed of necrotic lesions with residues of viable tumor cells observed in some areas. The liver background was A1/F4 (new Inuyama classification). The resection margins were negative, and R0 resection was confirmed. The patient was discharged 15 days after surgery without postoperative complications. There was no recurrence through 5 months after surgery.

## Discussion

We report a case of unresectable advanced HCC with PVTT in which atezolizumab plus bevacizumab therapy resulted in the regression of the PVTT and CS with R0 resection. To our knowledge, this is the first report on this specific therapeutic approach.

Hepatectomy is effective for HCC; however, PVTT is a strong indicator of poor postoperative prognosis. The surgical prognosis becomes worse as the tumor thrombus extends to the main trunk or contralateral branch of the portal vein [10]. The median survival time after surgery according to the extent of PVTT was as follows: 4.13 years for the third-order branch, 2.49 years for the second-order branch, 1.58 years for the first-order branch, and 0.19 years for the main trunk/contralateral branch. The prognosis for hepatectomy up to the first-order branch is better than that for non-liver resection. During surgery for HCC with PVTT, the portal vein containing the tumor thrombus is generally resected in combination with resection of the portal vein-dominated liver [11, 12]. However, this method often requires the resection of a large volume of liver and is difficult to perform for patients with poor liver function. In the present case, the tumor thrombus extended to the main trunk of the portal vein, making it difficult to perform R0 left lobectomy; a radical resection at that point would have entailed a high risk of postoperative complications, early recurrence, and a poor prognosis.

In contrast, several new agents with excellent antitumor effects have been approved for systemic chemotherapy in recent years, and CS for unresectable HCC is expected to increase in the future. Sorafenib has been the standard anticancer drug for HCC for a long time; although it prolonged overall survival (OS) (median OS of 10.7 months in the sorafenib group vs. 7.9 months in the placebo group) [1], the antitumor effect was weak and the success of subsequent CS was low. In 2018, the REFLECT trial showed that lenvatinib was not inferior to sorafenib based on OS, and was superior based on antitumor efficacy (18.8% response rate in the lenvatinib group vs. 6.5% in the sorafenib group) [2]. Lenvatinib followed by CS reportedly has an R0 resection rate of 8.4% [13] and there are several reports of CS cases [4–7]. In addition, in 2020, prolonged OS and superior antitumor efficacy of atezolizumab plus bevacizumab versus sorafenib were demonstrated in the IMbrave150 trial (median OS not reached in the atezolizumab plus bevacizumab group vs. 13 months in the sorafenib group; response rate 27% in the atezolizumab plus bevacizumab group vs. 12% in the sorafenib group) [3]. In response, the 2021 Clinical Practice Guidelines for HCC in Japan recommends atezolizumab plus bevacizumab for first-line treatment of unresectable HCC in the absence of autoimmune disease and sorafenib or lenvatinib for patients with autoimmune disease. To date, there is a limited number of case reports on CS with atezolizumab plus bevacizumab [8, 9]. Komatsu et al. reported a case of PVTT in the main trunk in which atezolizumab plus bevacizumab therapy resulted in a marked reduction of the PVTT [14]. In the present case, regression of the PVTT was also obtained, enabling R0 left lobectomy. Histopathological findings showed that most of the PVTT was necrotic, indicating the favorable therapeutic efficacy of atezolizumab plus bevacizumab for PVTT. The fact that atezolizumab plus bevacizumab did not cause a decrease in performance status or liver function was advantageous for the conduct of CS.

When R0 hepatectomy can be performed in CS, a good therapeutic prognosis can be expected. Shindou et al. performed a prospective analysis of patients with unresectable HCC who received lenvatinib therapy [13]. Twelve of 107 patients underwent CS with curative intent, and R0 resection was performed for 9 of 12 patients (R0 achievement rate: 8.4%). Furthermore, at a median observation period of 27.4 months, the median survival was 19, 8.9, and 11.1 months for the R0 resection group, R2 resection group, and no intervention group, respectively. The treatment prognosis was superior in the group that received lenvatinib followed by R0 resection with CS. There are several case reports of CS enabled by lenvatinib [4–7]. Among these cases, lenvatinib reportedly reduced the size of HCC with arterioportal shunt [4], HCC with preceding TACE treatment for its rupture [5], PVTT in the main trunk and contralateral branch [6], and intrahepatic recurrent HCC [7]. However, case reports on CS enabled by atezolizumab plus bevacizumab are limited [8, 9]. Wang et al. reported the first case of CS with atezolizumab plus bevacizumab. According to their report, 15 courses of atezolizumab plus bevacizumab therapy reduced the size of HCC but the patient later experienced disease progression and then underwent CS [8]. Hoshino et al. reported a case of HCC with adrenal metastasis and inferior vena cava invasion in which nine courses of atezolizumab plus bevacizumab therapy enabled CS, and pathological complete response was achieved [9]. R0 resection without postoperative recurrence was achieved in all of these CS case reports. In the present case, atezolizumab plus bevacizumab resulted in regression of PVTT, and the patient was successfully given CS with R0 resection without postoperative recurrence.

Good responses to immune checkpoint inhibitors are known to be associated with immune-related adverse events [15]. In the present case, a grade 3 skin disorder was observed as an immune-related adverse event caused by atezolizumab, and CS was performed instead of continuing the treatment. The histopathological findings demonstrated that primary tumor and PVTT were predominantly replaced with necrotic lesions. We thus consider that the patient was showing a good response to atezolizumab.

Bevacizumab has a suppressive effect on wound healing. The appropriate interval between the cessation of administration and surgery is unknown. The estimated half-life of bevacizumab is 20 days. Periods of 4 weeks [16] and 6–8 weeks [17] have been recommended for suspension prior to surgery. In the present study, we withheld bevacizumab for 4 weeks before surgery and there were no surgical-wound complications. However, we acknowledge that the appropriate interval should be determined for each individual case.

There is no consensus by experts on the appropriate timing of CS. The decision is made by the surgeon based on a combination of factors, including a reduction in AFP/DCP levels on blood tests, imaging findings that suggest R0 resection, general condition, liver function, and side effects of chemotherapy, as in the present case.

## Conclusions

Here, we describe a case of unresectable HCC with PVTT, which was successfully treated with CS after atezolizumab plus bevacizumab therapy. This therapeutic approach may enhance outcomes for patients with unresectable HCC.

## Data Availability

The datasets obtained during the current study are available from the corresponding author on reasonable request.

## Abbreviations

AFP: Alpha fetoprotein
CS: Conversion surgery
CT: Computed tomography
DCP: Des-gamma-carboxyprothrombin
HCC: Hepatocellular carcinoma
OS: Overall survival
PVTT: Portal vein tumor thrombus
RECIST: Response evaluation criteria in solid tumors
TACE: Transcatheter arterial chemoembolization
